# Older adults' needs and preferences for a nutrition education digital health solution: A participatory design study

**DOI:** 10.1111/hex.13923

**Published:** 2023-11-28

**Authors:** Ashlee Turner, Victoria M. Flood, Haley M. LaMonica

**Affiliations:** ^1^ Sydney School of Health Sciences, Faculty of Medicine and Health The University of Sydney Sydney New South Wales Australia; ^2^ University Centre for Rural Health (Northern Rivers), Faculty of Medicine and Health The University of Sydney Lismore New South Wales Australia; ^3^ Translational Research Collective, Faculty of Medicine and Health The University of Sydney Sydney New South Wales Australia

**Keywords:** ageing, education, nutrition, participatory design, technology

## Abstract

**Background:**

The global population is ageing rapidly and there is a need for strategies to promote health and wellbeing among older adults. Nutrition knowledge is a key predictor of dietary intake; therefore, effective educational programmes are urgently required to rectify poor dietary patterns. Digital health technologies provide a viable option for delivering nutrition education that is cost‐effective and widely accessible. However, few technologies have been developed to meet the unique needs and preferences of older adults.

**Objective:**

The aim of this study was to explore technology use among older adults and qualitatively determine the content needs and design preferences for an online nutrition education resource tailored to older adult consumers in Australia.

**Methods:**

Twenty adult participants aged 55 years and older (95% female) participated in one of four 2‐h participatory design workshops. In each workshop, prompted discussion questions were used to explore participants' technology use and preferences and to explore content needs and design preferences for an online nutrition education resource specific to older adults.

**Results:**

All participants were regularly using a range of different devices (e.g., smartphones, tablets and computers) and reported being comfortable doing so. Participants wanted a website that provided general nutrition information, practical advice and recipes. To enhance engagement, they sought a personalised resource that could be adjusted to suit their needs, included up‐to‐date information and allowed for easy sharing with others by exporting information as a PDF.

**Conclusions:**

Participatory design methods generate new knowledge for designing and tailoring digital health technologies to be appropriate and useful for the target audience. Specifically, older adults seek an online resource that has large and simple fonts with clear categories, providing them with practical advice and general nutrition information that can be personalised to suit their own needs and health concerns, with the option to export and print information into a paper‐based format.

**Patient or Public Contribution:**

Older adults actively participated in the development and evaluation process to generate ideas about potential features, functionalities, uses and practicalities of an online nutrition education resource.

## 1 INTRODUCTION

The global population is ageing rapidly. In Australia, one in six adults is aged 65 years and older and it is predicted that older people will make up almost one‐quarter of the population by 2066.^1^ Approximately 80% of this demographic are living with at least one chronic disease.^1^ This puts significant strain on public healthcare services, leading to increased social and economic pressure on the wider community.^2^ Poor diet is a key contributor to chronic disease burden. Despite government health promotion campaigns, a large proportion of the population continues to underconsume healthful foods, with only 8% of adults over the age of 65 meeting the daily recommendations for fruit and vegetable intake.^3^ Improved population health relies on changing behaviour, particularly dietary habits, which can be challenging to sustain. Consequently, there is a need for effective resources and interventions tailored to support dietary behaviour change among the ageing population.

Numerous factors influence behaviour and one's capacity for behaviour change. As a starting point, knowledge and understanding are fundamental prerequisites for changing health‐ and nutrition‐related behaviour.^4^ Alongside other determinants of dietary behaviour, including price, preference and access, several studies have highlighted the significance of increased nutritional knowledge in promoting behavioural changes.^5^ Individuals with a better understanding of what constitutes a healthy diet are more likely to incorporate fruits and vegetables in their diets.^6^ Thus, nutrition education programmes may serve as a crucial first step in supporting healthy eating habits, particularly in older adults who often exhibit lower levels of nutrition‐related knowledge compared to their middle‐aged counterparts.^7^, ^8^ Research has demonstrated the effectiveness of education programmes targeting older adults, leading to improved overall nutrition knowledge and dietary intake in this population.^9^, ^10^ However, these studies delivered information through traditional in‐person lectures, which may not be appropriate for older adults facing challenges in attending in‐person sessions due to physical limitations, transportation barriers, or for those residing in regional or remote areas where the availability of such interventions is limited.

Digital health technologies are inherently well placed as a viable option for delivering educational programmes in a nontraditional format, particularly in the increasingly digital age during and following the COVID‐19 pandemic. Importantly, digital health technologies that provide health information and deliver health care are becoming increasingly accessible. In 2018, 61.6% of older Australians aged over 65 years reported using the internet in the previous 3 months, and in 2020, 93% of older adults had internet access in their homes.^11^, ^12^ There have also been clear increases in the number of older adults who own and use various devices, including smartphones, tablets, laptops and desktop computers.^12^ Digital health technologies have proved to be invaluable in the health care sector for providing services that are relatively cheap, timely and efficient compared to traditional models of care, with similar impact.^13^, ^14^ A major advantage of digital health over traditional models of care includes the capacity to recruit and deliver services on a large scale, as well as remotely, thereby enabling more widespread involvement in major public health issues.

Digital health technologies are increasingly being utilised in the management and treatment of a range of health conditions, including diabetes,^15^ healthy eating habits,^16^ obesity,^17^ sleep,^18^ physical activity^19^ and depression and anxiety.^20^ However, few have been developed specifically for older adults, representing a largely untouched area for potential technologies to improve the health and wellbeing of older adults in Australia and globally. Importantly, there is a high level of interest and motivation to use digital health technologies to improve health and wellbeing, with a study reporting that almost 95% of participants in a study of older adults aged 50 years and older would find it beneficial to be able to access a website designed to support healthy ageing, including overall physical health and cognition, as well as self‐management of existing conditions.^21^ Despite the benefits of digital health technologies and reported motivation to engage, there are several challenges for use in older adults, particularly around a lack of confidence in their ability to use technology.^22^, ^23^ As such, it is essential that digital health technologies be designed and tailored with the interests of the older adult population in mind, considering their unique and varying needs.

The Medical Research Council's Framework for Complex Interventions outlines four phases of intervention research that follow an iterative approach: development, feasibility, testing and implementation.^24^ The initial development phase emphasises engagement of relevant stakeholders and target end‐users to determine the potential outcomes from the development and implementation in a real‐world setting. The use of participatory design methodologies within the development phase positions potential end‐users as empowered participants to independently explore their needs to shape development outcomes.^25^ As such, the aim of this study was to explore technology use among older adults and qualitatively determine the content needs and design preferences for an online nutrition education resource tailored to older adult consumers in Australia.

## 2 METHODS

This study was part of a larger project to determine needs and preferences for an online nutrition education resource and evaluate the acceptability and feasibility of a digital health resource for delivering nutrition education to older adults. This study employed an exploratory qualitative study design using participatory design methods and adhered to the Consolidated Criteria for Reporting Qualitative Research guidelines^26^ (see Supporting Information S1: File 1). The study protocol was approved by the University of Sydney Human Research Ethics Committee (2022/423) and participants gave informed electronic consent before participation.

### 2.1 Participants

Participants were eligible if they were aged 55 years and older, proficient in English, lived in Australia and had completed the required informed consent process. A total of 20 people (95% female) aged 55 years and older participated in this study. Aligning with our previous work, we defined older adults as aged ≥55 years as this represents the age range when addressing risk factors (e.g., cardiovascular disease, diabetes, obesity) is typically recommended^27^ due to the increasing prevalence and, in turn, burden of diet‐related chronic disease beginning from the age of 55 years. Participants were not reimbursed for their participation.

### 2.2 Recruitment strategies

The study was digitally advertised through Facebook and the JoinUs national research register. Advertisements on both platforms were targeted to potential participants who met the inclusion criteria (i.e., were aged 55 years or older and lived in Australia). Additional targeting was used for Facebook advertisements to identify potential participants with interests in human nutrition and healthy lifestyles. Interested participants were directed to a study‐specific webpage using REDCap (Research Electronic Data Capture) hosted at the University of Sydney,^28^, ^29^ where they were able to read detailed study information and provide informed consent. Potential participants were advised that participation was entirely voluntary and that if they agreed to participate, they could withdraw their consent at any time without being required to provide any reasons.

### 2.3 Data collection

Participatory design workshops are collaborative and inclusive sessions that involve various keyholders, including target end‐users, in discussions around the design and development of digital health technologies. The primary goal of participatory design is to ensure that the outcome meets the needs, preferences and expectations of the people who will use and interact with it.^25^ As a part of the initial development phase (per the Framework for Complex Interventions), participatory design workshops allow for user input to be considered in the process of designing new technologies before significant investments are made in formal development.^24^

A series of four group‐based participatory design workshops of approximately 2‐h duration, each with up to 10 participants, were conducted in September 2022 with older adult consumers. The aim of each workshop was to actively engage the older adult community in discussions about how technology may be used to deliver nutrition education specific to the Mediterranean diet. The research team developed a semistructured discussion guide that prompted extensive discussion around the potential utility of an online nutrition education resource for older adults, what kind of information it would need to include and how it might look or function. The discussion guide was designed to enable the sessions to follow an iterative process so that initially generated ideas could be further developed by participants in subsequent workshops (see Supporting Information S2: File 2). Two researchers with experience in qualitative research (A. T., H. M. L.) conducted the workshops via videoconferencing (Zoom Video Communications Inc.). Three workshops were recorded and transcribed verbatim into Microsoft Word by A. T. The fourth group was not recorded due to facilitator error, but both facilitators took detailed field notes to capture participant comments.

Under the same ethics protocol, participants were invited to engage in a user‐testing study of an existing nutrition education resource. Those who consented to participate in both aspects of the protocol (i.e., participatory design workshops and user testing) provided basic demographic information via an online survey hosted on REDCap, including age, sex, level of education and country of birth.

### 2.4 Data analysis

Descriptive demographic data are expressed as mean ± standard deviation (SD) for continuous data, and frequencies and percentages for categorical data. Established deductive thematic analysis techniques were used to analyse the transcriptions and notes from the workshops.^30^ A coding framework outlining all key concepts was developed to analyse qualitative data by A. T and H. M. L. Data were coded in NVivo software^31^ using this framework. Coding followed an established iterative process of reading, coding and exploring the pattern and content of coded data, followed by reflection and discussion. A. T. and H. M. L. developed coding labels and coded 25% of the data collectively, with any differences of opinion being resolved through discussion to reach a consensus. The remaining data was coded independently by A. T., with discussion with H. M. L. as needed. Aligning with the topics explored in the participatory design workshops, five themes were identified: (1) technology use among older adults, (2) flexible access, (3) information needs, (4) personalised experience and (5) content from a trusted source. All themes were checked against each other and back to the original data to ensure that all relevant references had been gathered. This process resulted in a coherent and consistent thematic framework.

## 3 RESULTS

### 3.1 Participant characteristics

A summary of participants' demographic characteristics is presented in Table 1. Participants were well‐educated, mostly female and ranged in age from 56 to 74 (63.3 ± 5.8) years.

**Table 1:** Demographic characteristics of participants.

Continuous variables	Mean	SD
Age (years)^a^	63.3	5.8

Categorical variables	*n*	%
Sex^b^		
Male	2	11.1
Female	16	88.9
Education level^a^		
Postgraduate	9	56.3
Bachelors	3	18.8
Advanced certificate/diploma	3	18.8
Secondary (Years 7–12)	1	6.3
Country of birth^a^		
Australia	12	75.0
United Kingdom	2	12.5
Other^c^	2	12.5

^a^ Data missing for *n* = 4.

^b^ Data missing for *n* = 2.

^c^ Includes El Salvador and Canada.

### 3.2 Findings

The main findings are organised into the five themes identified via the data analysis process. Table 2 provides an overview of the themes, corresponding subthemes, codes and illustrative quotes.

**Table 2:** Overview of qualitative data analysis process.

Theme	Subtheme	Illustrative quotes	Codes
Technology use among older adults	Perceived competence	‘I think the fact that we're all here [via Zoom] shows that we are reasonably competent with this’ (Workshop 3)	Comfort
	Preferred devices, websites and apps	‘If I want to go on that site and look in more detail, I go on my computer because it's a bigger screen’ (Workshop 1) ‘I suppose, because you can use [a smartphone] on the bus, or when you're waiting for a haircut, or something like that’ (Workshop 3)	Device preferences Types of websites/apps
	Barriers to technology use	‘Most older people I know, they're still struggling to use the mobile phone. And they tell me they don't even know how to text people, let alone look something up’ (Workshop 2) ‘Whereas other people might not even do that, you might have to print it off yourself, put it in an envelope, put a stamp on and put it in the mail’ (Workshop 1)	Poor user experience Lack of digital literacy Preference for nondigital Unwillingness to engage
	Facilitators of technology use	‘I would say that, like I'm retired, and I have been for a while, and say of fifty people I can think of only one who isn't tech savvy’ (Workshop 1)	Ease of use Digital literacy
Flexible access		‘The more detailed information, you'd probably sit down at home when you've got time, and you know, you can read the website. But in terms of accessibility for the recipes and the ingredients, I think maybe on the phone would be … an app would be easier’ (Workshop 2)	Mode of access Format (website vs. app) Mobile friendly
Information needs		‘I certainly think the nutrition basics would be important’ (Workshop 1) ‘I mean, another suggestion could be is If you do have a recipe, then as an after you know, what you can do with the leftovers, so maybe you could convert it to this or to that’ (Workshop 2)	General nutrition Practical advice Recipes Relationship between diet and health
Personalised experience		‘One size does not fit all, but you know that's for sure. I get annoyed when people you know, they lost thirty kilos on a keto diet Therefore everyone else should be on it. And you know someone else gains thirty kilos type of thing’ (Workshop 1)	Tailored to individual needs Recipe adjustment Substitutions Conversions Search engine capabilities
Content from a trusted source		‘Where to go for more information. [I] would want to see peer‐reviewed studies to demonstrate that it is evidence based, [there is] so much fad diet [and] misinformation out there’ (Workshop 4)	Regular updates Evidence base Integration with established sources References

#### 3.2.1 Theme 1: Technology use among older adults

Subtheme: *Perceived competence*. Participants reported being generally confident and comfortable using technology. Statements included, ‘I'm pretty comfortable with technology’ (Workshop 1) and ‘I have no trouble with it’ (Workshop 3). Another participant remarked that ‘I think the fact that we're all here [via Zoom] shows that we are reasonably competent with this’ (Workshop 3).

Subtheme: *Preferred devices, websites and apps*. When asked ‘what kinds of devices are you using to access the internet’, participants reported a broad range of devices, including desktop and laptop computers, smartphones and tablets; computers were the most frequently referenced device. Computers were used most often because participants had ‘a preference for a larger device’ (Workshop 3), particularly when they wanted to ‘go on a site and look in more detail’ (Workshop 1) because ‘it's a bigger screen’ (Workshop 1). Smartphones were the most frequently criticised device because ‘a mobile phone is really annoying if you're trying to read an article because it's too small’ (Workshop 1). However, others preferred to use a smartphone because ‘your phones [always] with you’ (Workshop 3) and ‘you can use it on a bus, or when you're waiting for a haircut’ (Workshop 3).

Participants were using a broad range of different apps and websites across their different devices. The most common types of apps being used fell into the ‘Health’ category, which included general health (e.g., ‘booking appointments on the my doc app’), fitness and nutrition. Social media apps were also common, including Facebook, Messenger, Twitter and Instagram. Websites were the most common source for health information, including international health organisations, such as the ‘World Health Organisation’ and government‐based websites, such as the ‘[Australian] Department of Health’. Other participants utilised search engines, most typically Google, to look for general information on health conditions including ‘pain management, sleep apnoea, arthritis and diabetes’. Participants were also searching for specific nutrition information and recipes using a range of websites, including ‘Noom’, ‘real food dietitians’, ‘dietdoctor.com’, ‘well nourished’, ‘quirky cooking’ and ‘CSIRO’.

Subtheme: *Barriers to technology use*. The most common barrier to adoption and ongoing engagement with technology, including websites and apps, was poor user experience. This included issues such as ‘things that are in a very small [and silly] font’ (Workshop 3), ‘bad spelling and grammar’ (Workshop 1)), ‘pale print on a white background’ (Workshop 1) and ‘poor quality images and videos’ (Workshop 2).

A lack of digital literacy was also identified as a key barrier to using and engaging with technology, particularly for older adults. Participants emphasised that this would be an important consideration for the development of an online resource. Participants also noted that older adults may have differing levels of digital literacy, depending on the device being used and the context or situation for use. The general consensus was that older adults tend to be more comfortable and ‘be able to sit at home with their computer’ (Workshop 2), but they ‘are not necessarily going to be tech savvy enough to be dealing with an app in the supermarket’ (Workshop 2).

Other barriers to technology use included a preference for nondigital sources of information and a general unwillingness to engage with digital technology. To address these potential barriers to technology use, participants commented that online resources should include an option to export information as a PDF, so they had the ‘ability to be able to print information, if that's what you wanted to do’ (Workshop 3). This would enable easy sharing of the information, giving users ‘the ability to forward things easily to other people, or make suggestions to your friends that you know are on certain diets’ (Workshop 3), particularly for those who ‘find that it's easier to read a hard copy’ (Workshop 3) or ‘who don't access things online’ (Workshop 3).

Subtheme: *Facilitators of technology use*. Ease of use was the most commonly identified facilitator of technology use, with participants reporting that they were more likely to continually engage with a website or app when they found it easy to use. In particular, this is related to digital tools that ‘[have] clear categories’ (Workshop 2), ‘open and download quickly’ (Workshop 2) and are easy to navigate with ‘everything on the same page’ (Workshop 3). To facilitate ease of use for the older adult population, participants emphasised a number of key design features, including ‘good font size’ (Workshop 2), ‘simple fonts that are easy to read’ (Workshop 2), ‘the right colours of fonts’ (Workshop 2) with ‘really good contrast’ (Workshop 3), ‘clear categories to make it easy to find stuff’ (Workshop 2) and ‘fewer clicks needed to get to where you want to be’ (Workshop 1).

Digital literacy was also identified as a facilitator to using technology. Participants commented that they and most others that they knew were able to comfortably use technology, stating that ‘of fifty people, I can think of only one who isn't tech savvy’ (Workshop 1).

#### 3.2.2 Theme 2: Flexible access

Most participants stated that they would prefer a nutrition education platform to be hosted on a website, rather than an app. It was suggested that a website would be more likely to reach a larger audience because ‘most people would go to a website, only a few people would go to an app (Workshop 3). For those who would prefer an app, they also saw the benefits of a website and suggested having the platform accessible from a website and an app, depending on the intended use—‘you could have the website with more information, but if you wanted a quick meal, you could have an app’’ (Workshop 2). For the more detailed educational information, participants wanted to ‘go on my computer because it's a bigger screen’ (Workshop 1) and ‘sit down at home when you've got time, and you know, you can read the website’ (Workshop 1). This was preferred to using a mobile phone because ‘a mobile phone is really annoying if you're trying to read an article because it's too small’ (Workshop 2). However, they commented that for ‘accessibility for the recipes and the ingredients, I think maybe on the phone would be… an app would be easier’ (Workshop 2), particularly when they are on the go at the supermarket. Therefore, to maximise usability, participants referenced the need for the platform to be accessible on a desktop computer as well as to be mobile friendly.

#### 3.2.3 Theme 3: Information needs

When discussing the specific content and type of information they would need from an online nutrition education platform, participants noted several things they would want to include: (1) *practical advice* (e.g., information on how to read food labels, meal planning guidance to aid with organisation, a list of seasonal produce, suggestions on what to do with leftovers from a meal, budget‐friendly ingredients and recipes, suggestions for what to order when eating out and specific options for each meal of the day); (2) *general nutrition information* (e.g., food groups, nutrient sources, foods to combine to maximise nutrient absorption, nutrition information panels on recipes and evidence on the link between diet and diseases such as diabetes and Alzheimer's disease); and (3) *recipes*.

#### 3.2.4 Theme 4: Personalised experience

The primary preferred functionality was *personalisation* (i.e., a tool that is designed to meet the needs and requirements of the consumer according to data entered by the consumer) with participants commenting that they ‘would like something tailored to the individual specific to their health concerns’ (Workshop 4) because ‘one size does not fit all’ (Workshop 1). This included being able to enter relevant data to receive feedback and specific advice or recipes based on their current dietary intake, health conditions (e.g., Hashimoto's, gluten intolerance) and food preferences (e.g., low carbohydrates).

Another component of personalisation that participants wanted was the ability to adjust recipes based on the number of serves required, ‘being able to adapt it for different sized families, so you know, if there's one or two of you … being able to moderate the recipe’ (Workshop 3). Participants also noted that they wanted a list of substitutions to account for individual allergies and food preferences with each recipe, ‘If you don't want dairy, try this. If you're allergic to nuts, try this instead. If you want to go low fructose, try this’ (Workshop 1).

Participants also liked the idea of having a search engine that could be used to find specific information or recipes depending on what ingredients they have at home or what is on sale at the supermarket. This was particularly important for older adults who may be on a pension and needing to keep food costs and wastage down, ‘when you're on a pension, that's the only way to go, and a lot of older people are on a pension. So you know, whatever is available, seasonal and well‐priced is what I base my menu on’ (Workshop 3).

#### 3.2.5 Theme 5: Content from a trusted source

Participants emphasised the need for a resource that included up‐to‐date information that was evidence‐based and came from a trusted source. Participants noted that there is a lot of ‘fad diet and misinformation out there” (Workshop 4) so the inclusion of references would be helpful. Participants liked the idea of articles that included external links for where to go for ‘more in‐depth information should people require it’ (Workshop 1) and ‘links to peer‐reviewed publications’ (Workshop 1). To ensure that users were aware of how up to date the information is, it was suggested that it would be useful if the resource included a ‘this information is up to date at X’ (Workshop 1) statement to maintain transparency.

## 4 DISCUSSION

This participatory design study aimed to explore technology use among older adults and to qualitatively determine the content needs and design preferences for an online nutrition education resource tailored to older adults in Australia. Given the rise of diet‐related disease burden among the ageing population, the provision of evidence‐based nutrition education may be an essential first step in supporting healthy eating habits in older adults. Traditional approaches to nutrition education often do not account for the unique needs of older adults, and digital health approaches present a viable alternative. However, there are limited technologies that have been developed to cater to the needs of older adults, and this is essential to address perceived barriers to the acceptance and adoption of digital health solutions before implementation.

Our findings on the use of technology are largely in line with previous data highlighting that older adults regularly use technology and feel comfortable using a range of different devices, including smartphones, computers and tablets.^32^ Consistent with the growth of smartphone ownership in older adults, all participants stated that they owned and regularly used smartphones. For example, smartphone ownership in the United States increased by 48% between 2012 and 2021 in adults over the age of 65, and 78% of Australians aged 50 years and over used a smartphone to access the internet in 2020.^12^, ^33^ Although many participants noted that they generally preferred to use other devices (i.e., laptop, tablet) depending on what they were doing (e.g., searching for information), others preferred using a smartphone because it is convenient and portable. Most participants stated that they would prefer a nutrition education resource to be hosted on a website, rather than an app. This is consistent with previous findings that older adults prefer accessing online health‐related information via a website as opposed to a smartphone app.^34^ Overall, the most common type of information being sought by participants was related to health, indicating that older adults are accepting of technology to access health‐related information. Furthermore, participants reported engaging with a range of nutrition‐specific sources, highlighting the acceptability of online sources of nutrition information.

Although it was not a personal concern among our participants, a lack of familiarity with technology has frequently been cited as a barrier to adoption for older adults. Participants did note that when thinking about their older friends and family members, a lack of digital literacy was a potential barrier to technology use and meaningful engagement. This speaks to the notion of a digital divide. Data from the United States shows that there is a notable difference in the ownership and use of smartphones and tablets between adults aged 65–69 years and those aged 75–79 years, with ownership rates ranging from 59% and 41% and 31% and 28%, respectively.^35^ Furthermore, individuals with higher education levels, specifically those with a bachelor's degree or higher, are significantly more likely to own a computer and/or smartphone.^21^ Therefore, it is essential to recognise the existence of this digital divide when examining technology use among older adults as they may face obstacles in accessing online information and resources. These obstacles have the potential to impede their ability to participate in online activities, such as accessing vital health information. Being able to download and print online information into hard‐copy formats is a common request from older adults,^36^ and participants suggested that being able to export and print information to give to others who lack digital literacy skills may be a solution to address potential barriers to technology use. Although issues related to digital exclusion were not personally relevant to them, due consideration should be given to these issues when delivering digital health technologies to the wider population.

The ultimate goal of nutrition education is to provide individuals with the knowledge and skills to consume a healthy diet. Importantly, designing nutrition education programmes based on a prior needs assessment with the target population has been shown to support nutrition‐related knowledge and behaviours among older adults.^37^ The data from this study identified a range of specific informational and content requirements for crafting a nutrition education resource tailored to promote an anti‐inflammatory dietary pattern. First, participants highlighted the need for general nutrition information, including details of what foods to prioritise, what foods to limit or avoid and the nutritional composition of foods (e.g., energy content). They also emphasised the need for practical advice, such as guidance on meal planning, using leftover ingredients, adapting recipes for smaller portion sizes, how to read nutrition panels on packaged foods and suggestions for what to order when eating out. This is a significant consideration, as practical nutrition knowledge and food skills—which encompass the wider components of meal preparation such as meal planning, grocery shopping and budgeting—are more closely linked to dietary behaviours than general nutrition knowledge.^38^, ^39^

Given that preparing and cooking meals at home is associated with improved diet quality,^40^ providing older adults with resources to facilitate this will not only allow them to actively apply their nutritional knowledge, but it will also promote the development of their cooking skills and boost their confidence in preparing healthy meals, which is likely to contribute to improving diet quality. Similar content requirements were identified in a sample of older adults in Germany using participatory design methods to design a nutrition chatbot, highlighting key similarities in the nutrition information needs of older adults across populations.^41^ Collectively, these findings emphasise the multifaceted informational needs of older adults and provide a valuable foundation for shaping the content of nutrition education programmes aimed at improving diet quality in this demographic.

Although participants highlighted specific informational and content needs, more importantly, they desired the information to be personalised and tailored to the individual. This need for personalised health information has been consistently reported in the literature.^32^ Furthermore, there is growing evidence to suggest that providing individuals with personalised feedback and advice is considerably more effective in prompting changes in dietary behaviours compared to generic, one size fits all information.^42^ Older adults are also likely to be more trusting and confident in educational materials that are tailored and specifically relevant to them as an individual.^36^ Personalised nutrition approaches offer individuals tailored dietary recommendations based on their unique characteristics, such as their current diet, health status (e.g., diagnosed conditions, blood glucose and cholesterol levels and food allergies) and personal preferences (such as taste preferences). In this context, digital health technologies are inherently well placed to deliver cost‐effective personalised nutrition advice in real‐time, and their feasibility and efficacy in improving dietary behaviours have already been demonstrated.^43^ Further research is needed to investigate the feasibility and effectiveness of providing personalised nutrition education and advice, specifically based on an anti‐inflammatory diet, to older adults via digital health technologies.

Written materials are a fundamental component of health‐related education, including nutrition education. Among various methods of health education delivery (e.g., group classes), written health information (including online and hard copy) is favoured by older adults^36^, ^44^ and it allows them the flexibility to engage with the content in their own time and at their own pace. While ensuring appropriate readability levels of written materials is crucial, the layout and presentation of the information are equally vital aspects that contribute to overall suitability and usability of materials and are often overlooked.^45^ Consistent with prior research,^36^, ^46^ participants noted several design and presentation principles to minimise issues with usability specific to the needs of older adults. This included the use of large and simple fonts, using dark type on a light background (i.e., black font on a white background) and incorporating clear categories to facilitate easy navigation. These suggestions represent just a fraction of the guidance available for developing effective written health education materials, drawing from the existing body of evidence.^47^ The findings from this study, alongside the broader spectrum of evidence, emphasise the importance for researchers and health professionals to consider and consult these recommendations when creating and evaluating written health information for older adults. Moreover, there is a need for further research aimed at identifying the most critical design and presentation principles for enhancing the usability and uptake of online written health information and how these elements contribute to facilitating behaviour change.

This study is not without its limitations. Our recruitment strategy exclusively relied on online recruitment methods. While online strategies offer an innovative avenue to reach a wide audience of potential participants, they tend to attract individuals who are more affluent and possess higher levels of education.^48^ This is consistent with the demographic profile of participants included in this study, where 94% of participants held postsecondary education qualifications (compared to approximately 31% of the general older adult population^1^). The sample is also relatively privileged in that most reported owning and using multiple different devices to access the internet. This constrained our study cohort to participants who regularly used and engaged with technology and reported being very comfortable using a range of different devices. Consequently, our study could not delve into the accessibility, engagement and appropriateness of technology for novice users or those without reliable access to technology. Future research should utilise a combination of online and offline recruitment channels in an effort to recruit diverse samples with differing levels of technology use and experience. Moreover, it is important to acknowledge that our sample is a self‐selected group of individuals who are likely to be more health conscious, and therefore willing to engage with health‐related research, than the general population. Therefore, it remains unclear to what extent these findings are applicable to the broader older adult demographic, and our capacity to generalise our findings is limited.

## 5 CONCLUSION

The purpose of this study was to build an understanding of the content needs and design preferences for an online nutrition education resource. Specifically, older adults seek an online resource that has large and simple fonts with clear categories, providing them with practical advice and general nutrition information that can be personalised to suit their own needs and health concerns, with the option to export and print information into a paper‐based format. Given the lack of online, self‐directed and evidence‐based nutrition education resources for older adults, the findings from this study can be used to design and develop digital health solutions that meet the needs and preferences of older adults. Although the proportion of older adults who use and engage with technology has increased over time, it is possible that the uptake of digital health solutions may be impacted by variations in digital literacy. Active co‐design of digital health tools with the target population may assist with increasing adoption and acceptance; however, we must also consider the need for ongoing education and digital literacy training.

## CONFLICT OF INTEREST STATEMENT

Victoria M. Flood is the President‐elect of the Nutrition Society of Australia, the Director of the Australian Rural Health Education Network and an Editor for the journals Nutrition & Dietetics and Nutrients. These are voluntary roles and no payment is received for the positions. The remaining authors declare no conflict of interest.

## ETHICS STATEMENT

This study was conducted according to the guidelines laid down in the Declaration of Helsinki and all procedures involving research study participants were approved by the University of Sydney Human Research Ethics Committee. Electronic informed consent was obtained from all participants.

## Supporting information

Supporting information.

Supporting information.

## Data Availability

The data that support the findings of this study are available from the corresponding author upon reasonable request.
