# Supplementary material for: Older adults' needs and preferences for a nutrition education digital health solution: A participatory design study
Source: Health Expect. 2023 Nov 28;27(1):e13923. doi: 10.1111/hex.13923 (PMC10734207; doi:10.1111/hex.13923)
Supplement: Supplementary file 1 — Supporting information. [file HEX-27-e13923-s002.docx]

**Supplementary File 1: Consolidated criteria for reporting qualitative research (COREQ) Checklist**

| **Domain 1: Research team and reflexivity** |  |
| --- | --- |
| *Personal Characteristics* |  |
| 1. Interviewer/facilitator: Which author/s conducted the interview or focus group? | HL – conducted 2 groups  AT – took notes during the first 2 groups and conducted the second 2 groups |
| 1. Credentials: What were the researcher’s credentials? E.g. PhD, MD | AT holds a BPsych(Hons) and MBMSc |
| 1. Occupation: What was their occupation at the time of the study? | AT is a PhD Candidate and Research Assistant |
| 1. Gender: Was the researcher male or female? | AT is a female |
| *Experience and training* |  |
| 1. What experience or training did the researcher have? | AT has organised and conducted >10 individual semi-structured interviews with research participants. She was trained on conducting focus groups by HL |
| *Relationship with participants* |  |
| 1. Relationship established: Was a relationship established prior to study commencement? | AT had no prior relationships with any of the adults who took part |
| 1. Participant knowledge of the interviewer: What did the participants know about the researcher? e.g. personal goals, reasons for doing the research | None of the participants knew the interviewer prior to the focus group |
| 1. Interviewer characteristics: What characteristics were reported about the interviewer/facilitator? e.g. Bias, assumptions, reasons and interests in the research topic | No characteristics were reported |
| **Domain 2: study design** |  |
| *Theoretical framework* |  |
| 1. Methodological orientation and Theory: What methodological orientation was stated to underpin the study? e.g. grounded theory, discourse analysis, ethnography, phenomenology, content analysis | Framework approach – thematic analysis |
| *Participant selection* |  |
| 1. Sampling: How were participants selected? e.g. purposive, convenience, consecutive, snowball | Convenience |
| 1. Method of approach: How were participants approached? e.g. face-to-face, telephone, mail, email | Advertisements were posted on social media and volunteer research registers |
| 1. Sample size: How many participants were in the study? | 44 consented and invited  20 included in final analysis |
| 1. Non-participation How many people refused to participate or dropped out? Reasons? | 21 did not respond  3 agreed to take part but did not attend due to various reasons (e.g. sickness) |
| *Setting* |  |
| 1. Setting of data collection: Where was the data collected? e.g. home, clinic, workplace | Online using Zoom teleconference |
| 1. Presence of non-participants: Was anyone else present besides the participants and researchers? | No |
| 1. Description of sample What are the important characteristics of the sample? e.g. demographic data, date Data collection | N/A – demographic data was not collected |
| 1. Interview guide: Were questions, prompts, guides provided by the authors? Was it pilot tested? | Questions were asked by the interviewer but were not provided to participants |
| 1. Repeat interviews: Were repeat interviews carried out? If yes, how many? | 4x focus groups were held until thematic saturation was reached |
| 1. Audio/visual recording: Did the research use audio or visual recording to collect the data? | Audio recording was used to collect data via Zoom teleconference |
| 1. Field notes: Were field notes made during and/or after the interview or focus group? | Yes, during and after |
| 1. Duration: What was the duration of the interviews or focus group? | 2 hours |
| 1. Data saturation: Was data saturation discussed? | Yes |
| 1. Transcripts returned: Were transcripts returned to participants for comment and/or correction? | No |
| **Domain 3: analysis and findings** |  |
| *Data analysis* |  |
| 1. Number of data coders: How many data coders coded the data? | Two data coders for 25% of data (AT and HL), AT coded remaining independently |
| 1. Description of the coding tree: Did authors provide a description of the coding tree? | Yes |
| 1. Derivation of themes: Were themes identified in advance or derived from the data? | Derived from the data |
| 1. Software: What software, if applicable, was used to manage the data? | Transcripts produced in Microsoft Word, thematic analysis in NVivo 12 |
| 1. Participant checking: Did participants provide feedback on the findings? | No |
| *Reporting* |  |
| 1. Quotations presented: Were participant quotations presented to illustrate the themes / findings? Was each quotation identified? e.g. participant number | Yes |
| 1. Data and findings consistent: Was there consistency between the data presented and the findings? | Yes |
| 1. Clarity of major themes: Were major themes clearly presented in the findings? | Yes |
| 1. Clarity of minor themes: Is there a description of diverse cases or discussion of minor themes? | Yes |
