# Supplementary material for: Older adults' needs and preferences for a nutrition education digital health solution: A participatory design study
Source: Health Expect. 2023 Nov 28;27(1):e13923. doi: 10.1111/hex.13923 (PMC10734207; doi:10.1111/hex.13923)
Supplement: Supplementary file 2 — Supporting information. [file HEX-27-e13923-s001.docx]

**PARTICIPATORY DESIGN WORKSHOP**

**DRAFT AGENDA**

*Study Title: Participatory design to inform a subsequent online nutrition education program for older adults*

**Welcome and workshop overview (15 minutes)**

- Introduction and housekeeping
- Overview of the workshop

**DISCOVERY (45 minutes):**

Exposure to the Mediterranean diet/nutrition education –

- Have you ever actively sought out nutrition information? Where do you go to find the information you want/need (e.g. dietitian/nutritionist, searching for info online, intervention study, etc)?
- Have you ever heard of the Mediterranean diet before?
- If yes, do you know anything about the diet (e.g. typical foods/meals to include/exclude, cooking methods)?

Technology use among older adults –

- How comfortable are you using technology and/or accessing the internet?
- If you use the internet, what sort of websites/services do you use?
- What kind of devices are you using to access the internet (e.g. computer, smartphone, iPad/tablet)? Use of apps versus websites?
- What turns you off when you look at a website (e.g. movement, colour, too much text, etc)? What brings you back to an app/website? What helps you to stay engaged (e.g. quality of info, games, necessity, etc)? Examples from participants of websites and/or apps that they use/like 🡪 provide examples of good/bad websites geared towards older adults for feedback on what’s good vs bad (e.g. Lumosity).
- Do you use technology to access health and wellbeing services or information? Have you ever engaged in any online health-related programs (e.g. online IQ tests, cognitive brain training, exercise, etc)? Engagement in telehealth (e-Health) health services (e.g. GP, dietitian, etc)? If yes, via phone, Zoom, Skype etc.

**BREAK (15 minutes)**

**PROTOTYPING (45 minutes):**

If there were no limitations, and any were possible, what would you want from a technology solution to provide nutrition education for healthy ageing?

- Consider: what kind of technology is it (e.g. app, website)? How do you want to access it (e.g. smartphone, computer)? What could it look like? Who is it for? When would they use it? What does it include? What kind of supports would be included (e.g. links to other resources/nutritionists/dietitians, recipes, cooking demos)?
- Would you want functionality to track food intake or general diet adherence, weight, other health outcomes?
- Would you like a reward system as a part of tracking the above? Reward for engaging with diet versus reward for success – what kind of goals would you set re improving food quality (goals re other health outcomes?)?
- Is this something you would be able to use independently, or share among family/friends/social group?
- Potential: show examples of different resources available (websites, brochures, etc) – what things do you like? What things do you not like?

**EVALUATION (30 minutes):**

- What other considerations need to be accounted for the older adult community?
- What would you see as some issues accessing this type of support online?
- Implementation – how would you want this implemented into your health care (independent, with a dietitian/other health professional)? Phone calls, text messages, etc.

**Wrap up and thoughts (10 minutes)**

- Questions
- Thoughts / feedback
